# Rate of promoter class turn-over in yeast evolution

**DOI:** 10.1186/1471-2148-6-14

**Published:** 2006-02-10

**Authors:** Georgii A Bazykin, Alexey S Kondrashov

**Affiliations:** 1 Department of Ecology and Evolutionary Biology, Princeton University, Princeton, New Jersey 08544, USA; 2 National Center for Biotechnology Information, NIH, Bethesda, Maryland 20894, USA

## Abstract

**Background:**

Phylogenetic conservation at the DNA level is routinely used as evidence of molecular function, under the assumption that locations and sequences of functional DNA segments remain invariant in evolution. In particular, short DNA segments participating in initiation and regulation of transcription are often conserved between related species. However, transcription of a gene can evolve, and this evolution may involve changes of even such conservative DNA segments. Genes of yeast *Saccharomyces *have promoters of two classes, class 1 (TATA-containing) and class 2 (non-TATA-containing).

**Results:**

Comparison of upstream non-coding regions of orthologous genes from the five species of *Saccharomyces *sensu stricto group shows that among 212 genes which very likely have class 1 promoters in *S. cerevisiae*, 17 probably have class 2 promoters in one or more other species. Conversely, among 322 genes which very likely have class 2 promoters in *S. cerevisiae*, 44 probably have class 1 promoters in one or more other species. Also, for at least 2 genes from the set of 212 *S. cerevisiae *genes with class 1 promoters, the locations of the TATA consensus sequences are substantially different between the species.

**Conclusion:**

Our results indicate that, in the course of yeast evolution, a promoter switches its class with the probability at least ~0.1 per time required for the accumulation of one nucleotide substitution at a non-coding site. Thus, key sequences involved in initiation of transcription evolve with substantial rates in yeast.

## Background

Comparison of long, orthologous DNA sequences usually reveals patterns consisting of alternating segments of higher and lower interspecies similarity [1]. Many slowly evolving segments are under selective constraint, due to their function as protein-coding exons, UTRs, transcription factor binding sites, etc. In particular, numerous relatively short conservative segments of untranscribed intergenic regions have recently been discovered, and phylogenetic footprinting has been used to study the molecular mechanisms of transcription [2-7].

However, functionally important DNA segments are not always strictly conserved, and can evolve due to a variety of factors, including positive selection [8-11]. This evolution leads to intraspecies polymorphism, often having significant impacts on function and fitness (reviewed in [12]), and to interspecies divergence. The known cases of such divergence usually involve presence of a functional binding site for a particular transcription factor in one species and its disruption or total absence in the orthologous sequence segment in the other species. Nucleotide substitutions, as well as short insertions and deletions involving a binding site, can be correlated with interspecies differences in the expression profiles of the corresponding genes [13-17].

Yeast *Saccharomyces *provides a particularly good opportunity to study evolution of functional segments of untranscribed DNA. In *S. cerevisiae*, and almost certainly in other related species, the promoter of a gene belongs to one of the two distinct classes: class 1 (TATA-containing) or class 2 (non-TATA-containing), with ~13% of all promoters containing a TATA box and belonging to class 1 [18]. Transcription from promoters of the two classes involves recruitment of different complexes of transcription factors [19], and the corresponding genes have rather different expression patterns. Expression of class 1 genes tends to change in response to selective pressure and environmental stress more than expression of class 2 genes [18,20]. Expression of class 1 genes is sensitive to mutations in binding surface of TBP, and their promoters often contain one of the eight variants of the 8 nucleotide-long TATA box consensus sequence [18]. TATA boxes are usually located in the region between 40 and 120 bp upstream of transcription start site [21,22]. Expression of class 2 genes is insensitive to mutations in binding surface of TBP, and their promoters usually lack TATA box consensus sequence [18].

Thus, evolution of a particular key transcription-related sequence, the TATA box, can be studied at the level of the whole yeast genome. Here, we will address the simplest, qualitative aspect of this evolution, the dynamics of switches of the promoter class in the course of interspecies divergence of orthologous genes within *Saccharomyces *sensu stricto group.

## Results

### Class 1 and class 2 promoters in *S. cerevisiae *genes

In order to study evolutionary switches of the promoter class, we first need to determine the class of individual genes. Let us start from considering *S. cerevisiae *genes where, in contrast to other yeast species, this task is facilitated by the available data on gene expression. Our goal is to establish two sets of genes, which unambiguously have class 1 or class 2 promoters in *S. cerevisiae*.

Although all genes apparently require TBP for expression, only a fraction of genes is sensitive to mutations in DNA binding surface of TBP [23], and these genes are inferred to have functional TATA boxes [18]. We assume that an ORF has a class 1 promoter in *S. cerevisiae *if it meets all of the following stringent criteria: (i) the upstream region (-180 to -70, relative to the ATG start codon) contains at least one TATA box consensus sequence, TATA(A/T)A(A/T)(A/G) [18], (ii) expression of the gene declined substantially (log_2 _ratio < -0.35) after 45 min exposure to at least one of the TATA binding defective TBP mutants V71E and V161E [18,23], and (iii) the location of the ORF on the chromosome does not overlap with that of any other ORF sensitive to TATA binding defective TBP mutants. These criteria define class 1 promoters with the highest possible certainty, as long as only *S. cerevisiae *sequence is used. Since we are interested in interspecies evolution of TATA box, our criteria must use exclusively the data from a single species *(S. cerevisiae) *and must not depend on interspecies sequence conservation [18]. A total of 212 (3.2%) *S. cerevisiae *ORFs meet these criteria; these are the genes that have class 1 promoters with the highest certainty.

Conversely, we assume that an ORF has a class 2 promoter in *S. cerevisiae *if it meets both of the following criteria: (i) the extended upstream region (-310 to -70, relative to the ATG start codon) does not contain any of the 8 TATA box consensus sequences, and (ii) expression of the gene was not affected (|log_2 _ratio| < 0.05) by 45 min exposure to both TATA binding defective TBP mutants V71E and V161E [18,23]. Among 397 genes that lack sensitivity to TBP mutations, 34 (8.6%) have a consensus TATA box sequence in their upstream regions (-180, -70) and another 41 have a consensus TATA box sequence in the (-181,-310) region. This leaves us with 322 (4.9%) *S. cerevisiae *ORFs which meet these criteria; these are the genes that have class 2 promoters with the highest certainty.

We concentrate on these two extreme classes of genes which very likely have class 1 or class 2 promoters and ignore the rest of the *S. cerevisiae *genes. Indeed, we have to focus on the extremes because they provide the strongest data sets.

### Class 1 and class 2 promoters in non-*cerevisiae *yeast genes

For the remaining 4 species of *Saccharomyces *sensu stricto group, there are no data on gene expression. Thus, we have to rely on sequences alone. We attribute to class 1 all the non-*cerevisiae *genes which carry at least one of the 8 TATA box consensus sequences in the (-180 to -70) region.

Dealing with class 2, we need to take into account that in *S. cerevisiae *a large fraction of genes sensitive to TBP binding defective mutations (198 out of 469, 42.2%) do not carry any of the 8 variants of the consensus TATA box sequences even in the extended upstream region (-310, -70). However, most of such genes (151 out of 198, 76.3%) carry a sequence differing by just one nucleotide from one of the variants. Therefore, some non-*cerevisiae *octanucleotides which are orthologous to a *S. cerevisiae *TATA box sequence but deviate at a single nucleotide site from the consensus still may function as TATA boxes, and the corresponding promoters may belong to class 1. Conversely, we assume that octanucleotides differing from each of the 8 TATA box consensus variants at two or more nucleotide sites do not function as TATA boxes, and attribute non-*cerevisiae *genes carrying only such sequences in their (-310, -70) regions to class 2.

### Promoter class switches between *S. cerevisiae *and other four species

**Figure 1:**
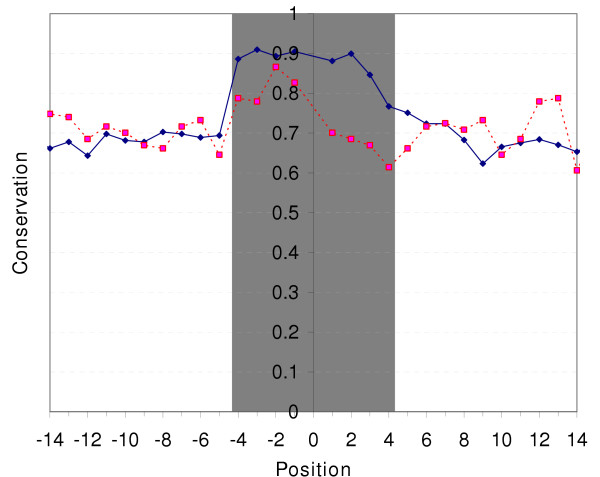
**Average per-nucleotide conservation of TATA box and of 10 nucleotides to its left and right**. Conservation of all four non-*cerevisiae *species is pooled together. Grey shading, TATA box; blue solid line, genes sensitive to mutations in DNA binding surface of TBP (N = 213); red dashed line, genes insensitive to mutations in DNA binding surface of TBP (N = 34).

**Figure 2:**
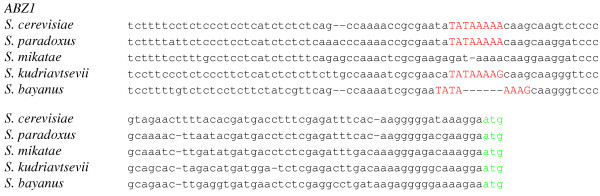
**Switch of promoter type by *ABZ1 *gene**. Red, TATA consensus sequence; green, ATG start codon. *S. cerevisiae *carries the consensus TATA(T/A)A(T/A)(T/G) sequence in position -73 relative to the ATG start codon. The consensus is also conserved in *S. paradoxus, S. kudriavtsevii *and *S. bayanus*. In *S. mikatae*, at least two nucleotides are substituted, eliminating the TATA box.

**Figure 3:**
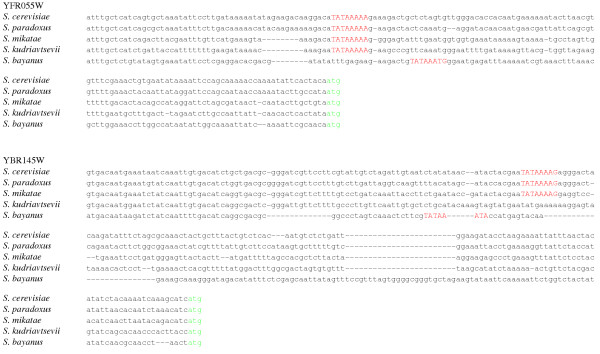
**Genes with functional class 1 (TATA box-containing) promoters in *S. cerevisiae *in which TATA box shifted in one of the other species of sensu stricto group**. Red, TATA consensus sequence; green, ATG start codon. In ORF YFR055W (hypothetical ORF), the distance in alignment between starts of TATA consensus sequences in *S. cerevisiae *and *S. bayanus *is 17 nucleotides. In ORF YBR145W (*ADH5*, alcohol dehydrogenase isoenzyme), the distance in alignment between starts of TATA consensus sequences in *S. cerevisiae *and *S. bayanus *is 19 nucleotides.

Generally, within the 212 sets of orthologous genes which very likely have class 1 promoters in *S. cerevisiae*, the putative TATA sites are strongly conserved, well above the level of conservation of surrounding sequence (chi-square, P < 0.0001; fig. 1). However, 17 (8.0%) of these genes lack a TATA box sequence (consensus or 1-nucleotide deviation) in one or more of the other four species from *Saccharomyces *sensu stricto group (Table 1). In these 17 genes, *S. cerevisiae *TATA box was aligned, in at least one of the non-*cerevisiae *species, either to an octanucleotide differing in more than one nucleotide from the consensus TATA box sequence, or to a gap (fig. 2). Promoters of 2 genes lack TATA box sequence in two or more non-*cerevisiae *species. The fraction of genes with missing TATA boxes is the lowest in *S. paradoxus*, the species most closely related to *S. cerevisiae *(Table 2). In 2 genes (0.9%), TATA box is present in *S. bayanus*, but its position differs from that in *S. cerevisiae*, and octanucleotides orthologous, in the *cerevisiae-bayanus *alignment, to both TATA boxes species differ by more than one nucleotide from the 8 consensus variants (fig. 3).

**Table 1:** Genes with class 1 (functional TATA box-containing) promoters in *S. cerevisiae *having orthologs which lack a TATA box in one or more other species of sensu stricto group.

		Description	Presence of TATA box^a^					
					
ORF	Gene name		*paradoxus*	*mikatae*	*kudriavtsevii*	*bayanus*	Switch events^b^	Ancestral state^c^
YLR109W	*AHP1*	Thiol-specific peroxiredoxin	+	+	+	- (2)	1	?
YPL221W	*BOP1*	Unknown function	+	+	+	- (2)	1	?
YBR298C	*MAL31*	Maltose permease	+	+	+	- (5)	1	?
YBR147W		Hypothetical ORF	+	+	- (2)	+	1	TATA
YCL035C	*GRX1*	Oxidoreductase	+	+	- (2)	+	1	TATA
YDR005C	*MAF1*	Mod5 protein sorting, negative effector of Pol III synthesis.	+	GA	- (2)	+	1	TATA
YPR193C	*HPA2*	Tetrameric histone acetyltransferase	+	+	- (2)	+	1	TATA
YDR533C	*HSP31*	Possible chaperone and cysteine protease	+	+	- (3)	+	1	TATA
YMR315W		Hypothetical ORF	+	+	- (3)	GA	1	?
YDR282C		Hypothetical ORF	+	- (2)	- (4)	+	2	?
YKL216W	*URA1*	Catalyzes the conversion of dihydroorotic acid to orotic acid	+	- (2)	+	+	1	TATA
YNR033W	*ABZ1*	Para-aminobenzoate (PABA) synthase	+	- (2)	+	+	1	TATA
YOR186W		Hypothetical ORF	+	- (2)	+	+	1	TATA
YOL143C	*RIB4*	Catalyzes synthesis of riboflavin	GA	- (3)	+	+	1	TATA
YPR119W	*CLB2*	Involved in mitotic induction	+	- (2)	GA	+	?	?
YLR346C		Unknown function	- (gap)	GA	+	+	1	TATA
YPL269W	*KAR9*	Karyogamy protein	- (gap)	- (gap)	- (gap)	- (gap)	1	non-TATA
Total			2	7	8	4		

^a ^For non-conserved TATA boxes, the minimal number of nucleotides different from the consensus sequence of TATA box (TATA(A/T)A(A/T)(A/G)) is shown in parentheses. "Gap" indicates an alignment gap at the site of the TATA box; "GA" means that ORF was not present in the given species.

^b ^Parsimonious number of switch events in sensu stricto group; i.e., the minimum number of mutations within sensu stricto group necessary to produce this pattern. The number of events is unknown for ORF YPR119W due to lack of data for *S. kudriavtsevii*.

^c ^Inferred state in last common ancestor. In cases when more than one equally parsimonious ancestral state was possible, it could not be inferred reliably (marked as '?').

**Table 2:** Evolution of class 1 (TATA box-containing) promoters between *S. cerevisiae *and other species of sensu stricto group

Species	Conserved ORFs	Switches of promoter type	Switches of promoter type per gene of this class per Ks^a^	TATA boxes shifted	TATA box shift events per TATA-containing gene per Ks^a^	Average conservation of upstream intergenic region
1. TBP-sensitive, TATA-containing genes (N = 212)						
*paradoxus*	200 (94.3%)	2	0.05	0	0.00	0.85
*mikatae*	180 (84.9%)	7	0.13	0	0.00	0.74
*kudriavtsevii*	179 (84.4%)	8	0.13	0	0.00	0.71
*bayanus*	178 (84.0%)	4	0.06	2	0.03	0.66
2. Non-TBP-sensitive, TATA-containing genes (N = 34)						
*paradoxus*	30 (88.2%)	8	1.40	0	0.00	0.79
*mikatae*	31 (91.2%)	10	1.08	0	0.00	0.73
*kudriavtsevii*	26 (76.5%)	14	1.58	0	0.00	0.65
*bayanus*	23 (67.6%)	9	1.09	2	0.24	0.63
3. Non-TBP-sensitive, non-TATA-containing genes (N = 322)						
*paradoxus*	278 (86.3%)	14	0.27	-	-	0.82
*mikatae*	241 (74.8%)	22	0.30	-	-	0.71
*kudriavtsevii*	218 (67.7%)	11	0.15	-	-	0.67
*bayanus*	238 (73.9%)	9	0.11	-	-	0.64

^a ^Number of events per time required for the accumulation of one nucleotide substitution at a non-coding site. The average number of substitutions per nucleotide site in intergenic regions, relative to *S. cerevisiae*, is 0.19 in *S. paradoxus*, 0.30 in *S. mikatae*, 0.34 in *S. kudriavtsevii*, and 0.36 in *S. bayanus*.

Among the 322 genes which very likely have class 2 promoters in *S. cerevisiae*, in 44 (13.7%) the consensus TATA sequence is present in (-180, -70) in one or more of the four non-*cerevisiae *yeast species. In 7 of these genes, it is present in two or more species (Tables 2, 3), and in 2 genes, it is present in all four species, providing strong support for its function as a TATA box in non-*cerevisiae *species.

**Table 3:** Genes with class 2 (non-TATA box-containing) promoters in *S. cerevisiae *having orthologs which have TATA box in one or more other species of sensu stricto group.

				Presence of TATA box					
						
ORF	Gene name	Description	Minimal distance from consensus^a^	*paradoxus*	*mikatae*	*kudriavtsevii*	*bayanus*	Switch events^b^	Ancestral state^c^
YDL139C	*SCM3*	Suppressor of chromosome missegregation	1	+	-	-	-		
YDR159W	*SAC3*	Component of nuclear pore	2	+	-	-	-	1	non-TATA
YGL091C	*NBP35*		1	+	-	-	-	1	non-TATA
YIR002C	*MPH1*	Helicase	1	+	-	-	-	1	non-TATA
YOL149W	*DCP1*	Decapping enzyme	1	+	-	-	-	1	non-TATA
YOR125C	*CAT5*		1	+	-	-	-	1	non-TATA
YLR011W	*LOT6*		1	+	GA	-	-	1	non-TATA
YOR154W		Hypothetical ORF	2	+	GA	-	-	1	non-TATA
YKL207W		Hypothetical ORF	1	+	GA	GA	GA	1	non-TATA
YDL005C	*MED2*	RNA Polymerase II transcriptional regulation mediator	4	-	+	-	-	1	non-TATA
YDL207W	*GLE1*	Polyadenylated-RNA-export factor	1	-	+	-	-	1	non-TATA
YDR459C			2	-	+	-	-	1	non-TATA
YER099C	*PRS2*	5-phospho-ribosyl-1(alpha)-pyrophosphate synthetase	2	-	+	-	-	1	non-TATA
YIL002C	*INP51*	Phosphatidylinositol 4,5-bisphosphate 5-phosphatase, synaptojanin-like protein	3	-	+	-	-	1	non-TATA
YNL125C	*ESBP6*		1	-	+	-	-	1	non-TATA
YOR201C	*PET56*	Ribose methyltransferase	1	-	+	-	-	1	non-TATA
YOR238W		Hypothetical ORF	5	-	+	-	-	1	non-TATA
YOR280C	*FSH3*	Serine hydrolase	1	-	+	-	-	1	non-TATA
YPL034W		Hypothetical ORF	2	-	+	-	-	1	non-TATA
YPL047W	*SGF11*		1	-	+	-	-	1	non-TATA
YPL096W	*PNG1*	De-N-glycosylation enzyme	2	-	+	-	-	1	non-TATA
YKL038W	*RGT1*	Transcriptional activator	4	-	+	GA	-	1	non-TATA
YDR422C	*SIP1*	Protein kinase complex component	1	-	+	-	GA	2	?
YOR211C	*MGM1*		3	-	+	-	GA	1	non-TATA
YPL112C	*PEX25*		2	-	+	GA	GA	1	non-TATA
YKL012W	*PRP40*	U1 snRNP protein involved in splicing	1	+	+	-	-	2	non-TATA
YGR134W	*CAF130*	CCR4 Associated Factor	1	+	+	-	GA	2	non-TATA
YBL074C	*AAR2*	Component of the U5 snRNP	1	-	-	+	-	1	non-TATA
YFR042W			2	-	-	+	-	1	non-TATA
YML065W	*ORC1*	Largest subunit of the origin recognition complex	4	-	-	+	-	1	non-TATA
YOR160W	*MTR10*		1	-	-	+	-	1	non-TATA
YOR228C		Hypothetical ORF	6	-	-	+	-	1	non-TATA
YPL091W	*GLR1*		1	-	-	+	-	1	non-TATA
YLR165C	*PUS5*		2	-	GA	+	-	1	non-TATA
YDR160W	*SSY1*	Component of the SPS plasma membrane amino acid sensor system (Ssy1p-Ptr3p-Ssy5p)	3	GA	-	+	GA	1	non-TATA
YBR108W		Hypothetical ORF	1	-	-	-	+	2	?
YHR105W	*YPT35*	Hypothetical ORF	2	-	-	-	+	1	?
YNL119W	*NCS2*		4	-	-	-	+	1	?
YKR053C	*YSR3*	Dihydrosphingosine 1-phosphate phosphatase	1	-	GA	GA	+	1	?
YOL020W	*TAT2*	Tryptophan permease	1	+	+	-	+	2	TATA
YMR169C	*ALD3*		1	-	+	GA	+	2	?
YMR170C	*ALD2*		1	-	-	+	+	1	TATA
YPR073C	*LTP1*		2	+	+	+	+	1	TATA
YDL054C	*MCH1*		5	+	+	+	+	1	TATA
Total				14	22	11	9		

^a ^The minimal distance from TATA consensus is the minimal number of nucleotides different from the consensus sequence (TATA(A/T)A(A/T)(A/G)) in the octanucleotide of *S. cerevisiae *which is orthologous to the TATA box in the non-*cerevisiae *species. "Gap" indicates an alignment gap at the site of the TATA box; "GA" means that ORF was not conserved in the given species.

^b ^Parsimonious number of switch events in sensu stricto group; i.e., the minimum number of mutations within sensu stricto group necessary to produce this pattern.

^c ^Inferred state in last common ancestor. In cases when more than one equally parsimonious ancestral state was possible, it could not be inferred reliably (marked as '?').

The known yeast phylogeny makes it possible to infer the promoter class of a gene in the last common ancestor of the *S. *sensu stricto group from the observed pattern of TATA box presence/absence in the five orthologs. Also, it is possible to use parsimony to infer the minimum number of promoter class switches (TATA box gain or loss) during the evolution of *S. *sensu stricto group species from their last common ancestor. In at least 7 of the 61 genes (11.5%) which underwent switches of the promoter class, there was more than one switch (Tables 1, 3).

Genes with promoters which switched their class between species are to some extent different from other genes of the corresponding class. The 44 class 2 (in *S.cerevisiae*) genes that switched class are generally expressed weaker in *S.cerevisiae *(Mann-Whitney U-test, U = 4991, P < 0.05) than the remaining class 2 genes. The 17 class 1 (in *S. cerevisiae*) genes that switched class are less sensitive to TBP mutations in *S. cerevisiae *(Mann-Whitney U-test, U = 826, P < 0.001) than the remaining class 1 genes.

Since 34 (out of 397) genes that lack sensitivity to TBP mutations in *S. cerevisiae*, have a consensus TATA box sequence in their (-180, -70) regions, even a perfect TATA box consensus may, nevertheless, fail to function as a TATA box, perhaps due to its broader sequence context. Conservation of such "spurious" TATA boxes between *S. cerevisiae *and other yeast species was substantially lower than for functional TATA boxes (chi-square, P < 0.0001; Table 2, fig. 1), and only slightly exceeded conservation of neighboring sequences (0.74 vs. 0.70, averaged over all four genomes; chi-square, P = 0.02; the slight excess of conservation was limited to the first four nucleotides of the TATA box (TATA)). The upstream regions of these 34 genes tend to be less conserved than that of the TBP-mutation-sensitive genes (chi-square, P < 0.0001; Table 2).

## Discussion

The sequence of the upstream region of a gene is not sufficient to determine the class of its promoter with perfect certainty. Some genes with expression profiles of class 1 genes nevertheless lack precise TATA boxes, and some genes with expression profiles of class 2 genes contain precise TATA boxes. Perhaps, sequences which deviate substantially from the TATA box consensus may act as TATA boxes in some class 1 genes, and TATA boxes in some class 2 genes are spurious. Alternatively, data on expression profiles of some genes might be problematic. Thus, at this point, we can regard class 1 genes as TATA-containing, and class 2 genes as non-TATA-containing, only with some degree of uncertainty. In the absence of the experimental data on gene expression in non-*cerevisiae *yeasts, our conclusions must be treated with caution, especially when applied to individual genes.

Our results suggest that even within the relatively short evolutionary times separating *Saccharomyces *species, a substantial fraction of genes underwent as major a transition as switching of the class of their promoters. At least 0.9% of all genes went through a switch of the promoter between the classes 1 and 2 at least once during the evolution of *Saccharomyces *sensu stricto group. This figure takes into account only the small fraction of genes for which we can determine the promoter class with the highest certainty. Extrapolated to the whole genome, this figure suggests that ~11% of genes change the class of their promoters in the course of sensu stricto group divergence.

Among genes belonging to class 1 in *S. cerevisiae*, the switch between promoter classes (either loss of TATA box in another species or gain of TATA box in *S. cerevisiae*) occurred at the rate of one per time during which eight to twenty substitutions occurred at a non-coding nucleotide site (Table 2). Conversely, among class 2 genes in *S. cerevisiae*, switches between promoter classes (either gain of TATA box in another species or loss of TATA box in *cerevisiae*) occur at the rate of one per time during which three to ten substitutions occurred at a non-coding nucleotide site (Table 2). Finally, at the rate of less than one per 50 nucleotide substitutions, the position of a TATA box shifted within the upstream region of a class 1 gene (Table 2).

The observed switches of promoter class cannot be due to sequencing errors. For example, a switch from class 1 in *S. cerevisiae *to class 2 in a non-*cerevisiae *yeast involves at least two nucleotide substitutions within the 8 nucleotide-long TATA box. Such switches were observed for 8% of TATA boxes, which would require a clearly impossible sequencing error rate >10^-2^.

Our estimates of the frequency of promoter class switches may be too low, for several reasons. First, we used very conservative definitions of class 1 and class 2 promoters. For the former, we required the presence of a TATA box within a narrow segment of the gene upstream region, and a significant reduction of the gene expression in TBP mutants. In reality, functional TATA boxes may be present in a broader region [24], and may be less sensitive to TBP mutations [18]; the latter is in fact the case for two of the genes experimentally known to contain a functional TATA box – *GAL1 *and *ADH1 *[19]. By limiting ourselves to the genes with the strongest response to mutation in TBP, we may be choosing a slowly evolving subset of TATA-containing genes. However, expression of genes with class 1 promoters can evolve rapidly in experiments [18], so that the real pattern may be more complex.

Second, for a fraction of the analyzed genes, we were unable to find the ortholog in another species. Since the five species considered are closely related, this can be due to the quality of the draft sequence. In reality, some of these yet undiscovered orthologous genes could have switched the classes of their promoters as well, and the rate of promoter type evolution inferred from better-quality genome sequences might be higher. Finally, our requirement of a change of two nucleotides in the ortholog of a TATA box may sometimes be too stringent, since a single nucleotide substitution is often sufficient to disrupt a functional TATA box [18,19] Therefore, the higher rate of promoter class switches inferred for the genes insensitive to TBP mutations with class 2 promoters in *S. cerevisiae *may reflect the actual rate of evolution better than the reciprocal rate.

The abundance of genes showing multiple events of promoter class switching during the evolution of *Saccharomyces *suggests heterogeneity of intrinsic switch rates among genes. In the genes with elevated rate of promoter class switching, TATA box can be under reduced selective constraint, or subject to fluctuating positive selection [25].

## Conclusion

By combining expression data on *S. cerevisiae *genes with sequence data from four closely related yeast species, we were able to ascertain the set of genes that probably changed the class of their promoters, and several genes in which the functional TATA box changed its position in the upstream region of the gene. Experimental data on non-*cerevisiae *genes are necessary to confirm our analysis for each individual gene. However, our results suggest that a substantial number of genes underwent promoter class switching between the closely related species of *Saccharomyces *genus.

## Methods

We used yeast genome annotation extracted from SGD database [26] to map 6578 ORFs on the finished genome of *S. cerevisiae *[27]. The genome of *S. cerevisiae *was aligned to draft genomes of another four species of *Saccharomyces *sensu stricto group (*S. paradoxus, S. mikatae, S. bayanus *[5] and *S. kudriavtsevii *[6]) using MLAGAN program [28] as described in [29]. To improve the quality of local alignments, each upstream region was re-aligned using ClustalW [30]. The alignments of the upstream regions for all the considered ORFs are available at [31]. For comparison with a non-*cerevisiae *species, we use only those *S. cerevisiae *ORFs which were aligned to an unambiguous ortholog in this species. Orthology was established according to the reciprocal best hits approach, using gapped BLAST [32]. In order to avoid possible complications due to large-scale genome rearrangements, we also required that the orthologs reside in syntenic region in the two genomes. For this purpose, we manually curated each region of alignment using OWEN program [33] and used only those pairs of orthologous ORFs which are embedded into long, continuous alignment which include, in particular, the ORF upstream to the considered pair of genes.

For each ORF in *S. cerevisiae*, we also check if its orthologs in another species remain functional, using the reading frame conservation test described in [5]. We assume that a reading frame was conserved in a given species if the maximum (for each of the three reading frames) percentage of in-frame nucleotides exceeds the threshold of 80% in *S. paradoxus*, 75% in *S. mikatae*, 70% in *S. kudriavtsevii *and 65% in *S. bayanus *[5]. We remove from further analysis those ORFs that were conserved in less than two non-*cerevisiae *species according to these criteria, since these are likely to be spurious ORFs [5]. In order to ensure that our position criteria for non-*cerevisiae *species are meaningful, we also require conservation of starting ATG codon in alignment in each considered species.

For the remaining pairs of unambiguous conserved orthologous ORFs, we analyze evolution of TATA box consensus sequences in their upstream regions. For 212 genes that certainly have TATA-containing class 1 promoters in *S. cerevisiae*, we analyze the fate of the TATA box in the other four species. We assume that a TATA box is conserved and its position remains invariant in a given non-*cerevisiae *species if the TATA box in *S. cerevisiae *sequence was aligned to the segment of the non-*cerevisiae *sequence which coincided with one of the 8 variants of the TATA-box consensus (nucleotide substitutions which kept the segment within the set of 8 variants of the TATA box consensus were allowed), or deviated from one of these 8 variants by no more than one nucleotide substitution.

Conversely, a fraction of non-*cerevisiae *sequence segments aligns to a consensus TATA box sequence in *S. cerevisiae*, but deviates from any of the consensus sequences by two or more nucleotides. In such cases, we assumed that the non-*cerevisiae *gene has a shifted TATA-box if one of the eight consensus variants was found elsewhere in the upstream region (-310 to -70). Alternatively, we assumed that a TATA box was missing if there was no TATA consensus sequence in this region.

Analogously, we analyze the evolution of certainly non-TATA-containing promoters in *S. cerevisiae*. If one or more of the eight exact TATA box sequences was present within the region (-180 to -70) in non-*cerevisiae *species, we assumed the promoter to be class 1 in this species.

In a few cases of ambiguous alignments of upstream regions, we were unable to tell with confidence which octanucleotide in one species was orthologous to TATA box in another. In such cases, we selected the octanucleotide with closest resemblance to TATA-box (i.e., with fewest nucleotides different from consensus TATA sequence) between two framing regions of unambiguous alignment, and considered it to be the ortholog of the TATA box. This approach was conservative in that it could only increase the actual conservation of the TATA box.
